# Supplementary material for: Mindfulness-Based Movement Intervention to Improve Sleep Quality: A Meta-Analysis and Moderator Analysis of Randomized Clinical Trials
Source: Int J Environ Res Public Health. 2022 Aug 18;19(16):10284. doi: 10.3390/ijerph191610284 (PMC9408303; doi:10.3390/ijerph191610284)
Supplement: Supplementary file 1 [file ijerph-19-10284-s001.zip › Search strategy.pdf]

Search Strategy

Mind Body Therapies OR Mind-Body Therapy OR Therapies, Mind-Body OR Therapy, Mind-Body OR Mind-Body Medicine OR Mind Body Medicine OR mind body OR Mindfulness OR mindfulness-based stress reduction (MBSR) OR mindfulness-based cognitive therapy (MBCT) OR Vipassanā OR Meditation OR Transcendental Meditation OR Meditation, Transcendental OR Yoga OR Tai Ji OR Tai-ji OR Tai Chi OR Chi, Tai OR Tai Ji Quan OR Ji Quan, Tai OR Quan, Tai Ji OR Taiji OR Taijiquan OR T'ai Chi OR Tai Chi Chuan OR Qigong OR Qi Gong OR Ch'i Kung OR Pilates OR Movement Techniques, Exercise OR Exercise Movement Technics OR Pilates-Based Exercises OR Exercises, Pilates-Based OR Pilates Based Exercises OR Pilates Training OR Training, Pilates AND sleep OR Sleeping Habits OR Sleep Habits OR Habit, Sleep OR Habits, Sleep OR Sleep Habit OR Sleeping Habit OR Habit, Sleeping OR Habits, Sleeping AND randomized controlled trial OR Randomized OR Placebo OR RCT
