# Supplementary material for: Mindfulness-Based Movement Intervention to Improve Sleep Quality: A Meta-Analysis and Moderator Analysis of Randomized Clinical Trials
Source: Int J Environ Res Public Health. 2022 Aug 18;19(16):10284. doi: 10.3390/ijerph191610284 (PMC9408303; doi:10.3390/ijerph191610284)
Supplement: Supplementary file 1 [file ijerph-19-10284-s001.zip › Sleep measure scores of included studies.pdf]

Sleep measure scores of included studies

Author	Year	Intervention Group Number	Intervention Baseline Mean	Intervention Baseline Standard	Intervention Final Mean	Intervention Final Standard	Control Group Number Control	Control Baseline Mean	Control Baseline Standard	Control Final Mean	Final Standard
Maryanna Klatt et al.	2017	27.00	5.93	1.80	3.89	1.60	30.00	5.13	2.28	4.83	2.67
Rshikesan et al.	2018	37.00	5.08	2.75	4.16	2.03	35.00	4.91	2.50	4.23	2.45
Curi et al.	2018	31.00	6.32	3.16	5.61	2.93	31.00	7.07	5.38	7.60	5.77
Özkan and Rathfisch	2018	42.00	7.40	4.03	3.31	1.96	42.00	6.83	3.27	8.74	2.80
McQuade et al.	2017	21.00	6.85	0.76	5.16	0.52	24.00	6.58	0.69	5.77	0.50
Aksu et al.	2018	13.00	2.91	1.55	4.39	1.76	13.00	4.15	3.18	9.85	1.99
Kerekes et al.	2017	77.00	9.70	4.60	8.10	3.90	75.00	10.60	5.10	10.00	4.80
Seyedi Chegeni et al.	2018	45.00	6.33	3.41	3.37	2.36	46.00	6.41	3.12	5.58	2.94
Zhu et al.	2018	42.00	5.03	2.55	3.32	1.92	38.00	6.08	3.06	4.50	2.51
Irwin et al.	2018	45.00	11.50	0.50	8.20	0.40	45.00	11.20	0.40	7.30	0.40
Chaoul et al.	2018	74.00	7.80	3.70	7.30	3.60	85.00	8.10	4.20	8.10	4.40
Ebrahimi et al.	2017	13.00	14.40	5.92	3.73	3.49	11.00	13.91	5.52	13.27	5.58
Hariprasad et al.	2013	62.00	7.65	3.36	6.87	3.10	73.00	8.19	3.46	8.78	3.77
Sun et al.	2013	38.00	9.62	3.50	6.30	2.97	37.00	9.50	3.79	10.84	3.76
Black et al.	2014	24.00	10.20	1.70	7.40	1.90	25.00	10.20	1.80	9.10	2.00
Zhang et al.	2015	30.00	11.50	3.28	8.17	2.61	30.00	11.27	3.62	11.47	3.58
Chan et al.	2016	27.00	10.20	3.00	7.80	3.30	25.00	9.80	3.10	9.60	2.40
Chandwani et al.	2014	53.00	8.30	0.60	6.70	0.50	54.00	8.20	0.50	7.30	0.50
Ashrafinia et al.	2013	40.00	9.97	2.21	5.45	1.51	40.00	10.40	2.68	8.35	1.83
Fang and Li	2015	61.00	9.98	1.89	7.61	1.25	59.00	10.24	2.35	10.31	2.42
Chan et al.	2014	75.00	10.00	3.70	8.20	3.40	75.00	10.20	3.80	9.50	3.70
Blake et al.	2016	63.00	6.23	2.51	4.79	1.97	60.00	6.39	2.83	5.93	2.32
Parco M. Siu et al.	2021	105.00	11.30	2.90	7.90	3.80	110.00	11.40	3.20	10.90	3.50
Shree Ganesh H.R. et al.	2021	48.00	10.29	5.32	10.23	4.60	48.00	13.15	7.64	19.77	9.55

Sleep measure scores of included studies

Phenphop Phansuea et al.	2020	33.00	10.00	5.14	4.82	2.30	33.00	7.79	4.22	6.94	3.59
Marian E. Papp et al.	2020	19.00	7.16	4.16	6.89	3.74	17.00	7.71	5.39	7.12	5.25
Zülfünaz Ozer and Sebahat Ates	2021	33.00	10.27	2.45	2.61	1.62	34.00	10.74	1.64	10.76	1.65
Jennifer L. Gordon et al.	2021	52.00	6.40	3.40	8.10	0.20	52.00	5.80	3.30	8.70	0.20
Keyla de Paula Barbosa et al.	2021	20.00	10.20	4.89	8.35	5.12	20.00	10.85	4.28	11.95	4.59
Fan et al.	2019	67.00	9.80	3.60	6.60	3.90	72.00	9.20	3.40	9.30	4.70
Agustín et al.	2019	55.00	8.56	4.98	7.16	4.90	52.00	7.10	4.42	8.38	4.28
Michael et al.	2019	19.00	8.07	3.97	6.80	4.23	17.00	6.19	3.33	6.19	3.33
Bai et al.	2019	78.00	7.73	2.95	6.62	2.25	78.00	7.46	3.09	7.41	2.98
Lu et al.	2019	43.00	10.51	1.45	4.10	1.10	44.00	10.45	2.13	6.90	2.00
Liu et al.	2019	45.00	11.30	3.10	7.50	2.20	46.00	11.20	2.70	10.50	2.40
Cetin et al.	2020	17.00	6.28	2.97	3.57	1.82	14.00	5.35	2.97	4.35	2.73
Habibollahpour et al.	2019	38.00	13.39	2.52	8.95	2.19	37.00	13.19	2.21	13.11	2.18
Gulhan et al.	2019	25.00	8.40	2.94	5.76	1.88	25.00	6.84	2.67	6.56	2.71
Bower, Julienne E et al.	2012	16.00	9.20	3.30	8.10	2.50	15.00	9.10	3.50	7.70	2.60
Chen, K. M et al.	2010	31.00	5.00	3.67	3.48	2.95	24.00	4.88	4.00	7.21	4.32
Chen, Kuei-Min et al.	2009	62.00	4.65	3.16	3.34	3.01	66.00	5.47	3.41	6.38	3.01
Frye, B et al.	2011	23.00	6.40	3.84	4.90	2.59	21.00	6.20	5.04	7.00	5.07
Lynch, Mary et al.	2012	53.00	13.79	2.95	10.50	3.94	47.00	13.09	3.79	12.47	3.02
Nguyen, Manh Hung and Kruse, Andreas	2012	48.00	9.38	4.99	3.59	1.56	48.00	8.06	4.09	7.97	3.78
Wang, W. C et al.	2010	16.00	7.81	4.13	5.13	3.36	13.00	7.00	4.24	6.15	3.48
Wolever, Ruth Q et al.	2012	90.00	7.69	0.35	5.22	0.34	53.00	7.38	0.46	6.02	0.44
Gregoire et al.	2017	68.00	12.76	6.91	11.05	6.26	24.00	10.54	6.73	12.00	5.54

Sleep measure scores of included studies

Duman and Timur Taşhan	2018	81.00	14.03	3.40	7.09	3.40	80.00	14.35	3.20	13.66	4.30
Zupanec et al.	2017	11.00	456.00	105.00	498.00	65.00	9.00	488.00	36.00	495.00	59.00
Greeson et al.	2014	45.00	41.36	16.21	32.52	18.06	45.00	42.91	16.21	42.84	17.23
Emel Sonmezer et al.	2021	20.00	28.46	22.76	15.79	14.18	20.00	28.98	21.99	28.70	21.77
Di Shao et al.	2021	72.00	7.81	4.64	5.70	3.35	72.00	6.76	3.66	6.78	3.78
Sanghee Moon et al.	2020	8.00	19.30	12.50	11.00	6.70	9.00	19.90	9.40	14.60	3.70
AKODU AK et al.	2021	14.00	11.07	5.08	4.64	2.98	14.00	12.00	4.37	6.64	3.23
Zhang et al.	2019	35.00	17.22	4.42	10.82	5.68	35.00	16.20	3.62	15.42	5.90
de Bruin et al.	2020	26.00	41.91	6.86	36.01	7.36	28.00	42.33	7.48	40.00	7.22
Biegel, Gina M et al.	2009	50.00	4.82	1.23	4.65	1.25	52.00	4.56	1.14	3.56	1.16
Chan, A. S et al.	2012	17.00	2.63	2.09	1.13	1.26	16.00	1.88	1.46	2.81	2.01
Chan, A. S et al.	2012	17.00	6.21	2.05	7.00	2.54	16.00	6.23	2.47	5.57	2.37
Chattha, R et al.	2008	54.00	0.74	0.99	0.44	0.72	54.00	0.85	0.98	0.74	0.91
Yurtkuran, M et al.	2007	20.00	4.60	3.60	3.40	5.20	20.00	4.60	3.70	4.30	8.20
