# Supplementary material for: Mindfulness-Based Movement Intervention to Improve Sleep Quality: A Meta-Analysis and Moderator Analysis of Randomized Clinical Trials
Source: Int J Environ Res Public Health. 2022 Aug 18;19(16):10284. doi: 10.3390/ijerph191610284 (PMC9408303; doi:10.3390/ijerph191610284)
Supplement: Supplementary file 1 [file ijerph-19-10284-s001.zip › Table S1. Egger's test with pseudo 95% confidence limits..pdf]

Table S1. Egger's test with pseudo 95% confidence limits.

Std Eff	Coef.	std. Err.	t	P>|t|	[95% Conf. Interval]	
slope	-.28426	.3439116	-0.83	0.412	-.9724256	.4039056
bias	-1.907378	1.443505	-1.32	0.191	-4.795825	.9810681
