# Supplementary material for: Mindfulness-Based Movement Intervention to Improve Sleep Quality: A Meta-Analysis and Moderator Analysis of Randomized Clinical Trials
Source: Int J Environ Res Public Health. 2022 Aug 18;19(16):10284. doi: 10.3390/ijerph191610284 (PMC9408303; doi:10.3390/ijerph191610284)
Supplement: Supplementary file 1 [file ijerph-19-10284-s001.zip › Table S2 - S7. Moderator analysis..pdf]

Table S2. Subgroup analysis based on the health status of the study population

Study group	Study N	Participants N	I-V pooled SMD	[95% Conf. Interval]		% Weight	*P value*
Health population	22	1063	-0.899	-1.000	-0.798	35.03	*p* <0.001
Clinical patients with physical illnesses	31	1086	-0.627	-0.736	-0.519	30.52	*p* <0.001
Psychiatric patients	19	973	-0.618	-0.720	-0.516	34.45	*p* <0.001

Table S3. Subgroup analysis based on the age of participants

Study group	Study N	Participants N	I-V pooled SMD	[95% Conf. Interval]		% Weight	*P value*
Younger adults	19	921	-0.618	-0.715	-0.520	39.67	*p* <0.001
Middle-aged adults	20	755	-0.691	-0.803	-0.579	29.93	*p* <0.001
Older adults	18	703	-0.873	-0.985	-0.761	30.40	*p* <0.001

Table S4. Subgroup analysis based on MBMI types

Study group	Study N	Participants N	I-V pooled SMD	[95% Conf. Interval]	% Weight	*P value*
Yoga/Pilates	25	1076	-0.808	-0.901 - 0.715	40.98	*p* <0.001
Tai Chi/Qigong/Baduanjin	18	682	-0.567	-0.680 - 0.455	28.35	*p* <0.001
MBMI combined with mindfulness or muscle relaxation techniques	18	756	-0.741	-0.849 - 0.633	30.67	*p* <0.001

Table S5. Subgroup analysis based on the duration of intervention

Study group	Study N	Participants N	I-V pooled SMD	[95% Conf. Interval]		% Weight	*P value*
≤3 months	48	1943	-0.714	-0.784	-0.644	73.45	p <0.001
>3 months	14	659	-0.829	-0.945	-0.712	26.55	p <0.001

Table S6. Subgroup analysis based on intervention frequencies

Study group	Study N	Participants N	I-V pooled SMD	[95% Conf. Interval]		% Weight	*P value*
≤1/week	15	647	-0.687	-0.804	-0.570	29.2	p <0.001
>1/week	40	1594	-0.793	-0.868	-0.718	70.71	p <0.001

Table S7. Subgroup analysis based on total intervention hours

Study group	Study N	Participants N	I-V pooled SMD	[95% Conf. Interval]		% Weight	*P value*
≤24 h	35	1394	-0.746	-0.827	-0.665	63.34	*p* <0.001
> 24 h	17	767	-0.759	-0.865	-0.653	36.66	*p* <0.001
