# Supplementary figures and images for: Mindfulness-Based Movement Intervention to Improve Sleep Quality: A Meta-Analysis and Moderator Analysis of Randomized Clinical Trials
Source: Int J Environ Res Public Health. 2022 Aug 18;19(16):10284. doi: 10.3390/ijerph191610284 (PMC9408303; doi:10.3390/ijerph191610284)

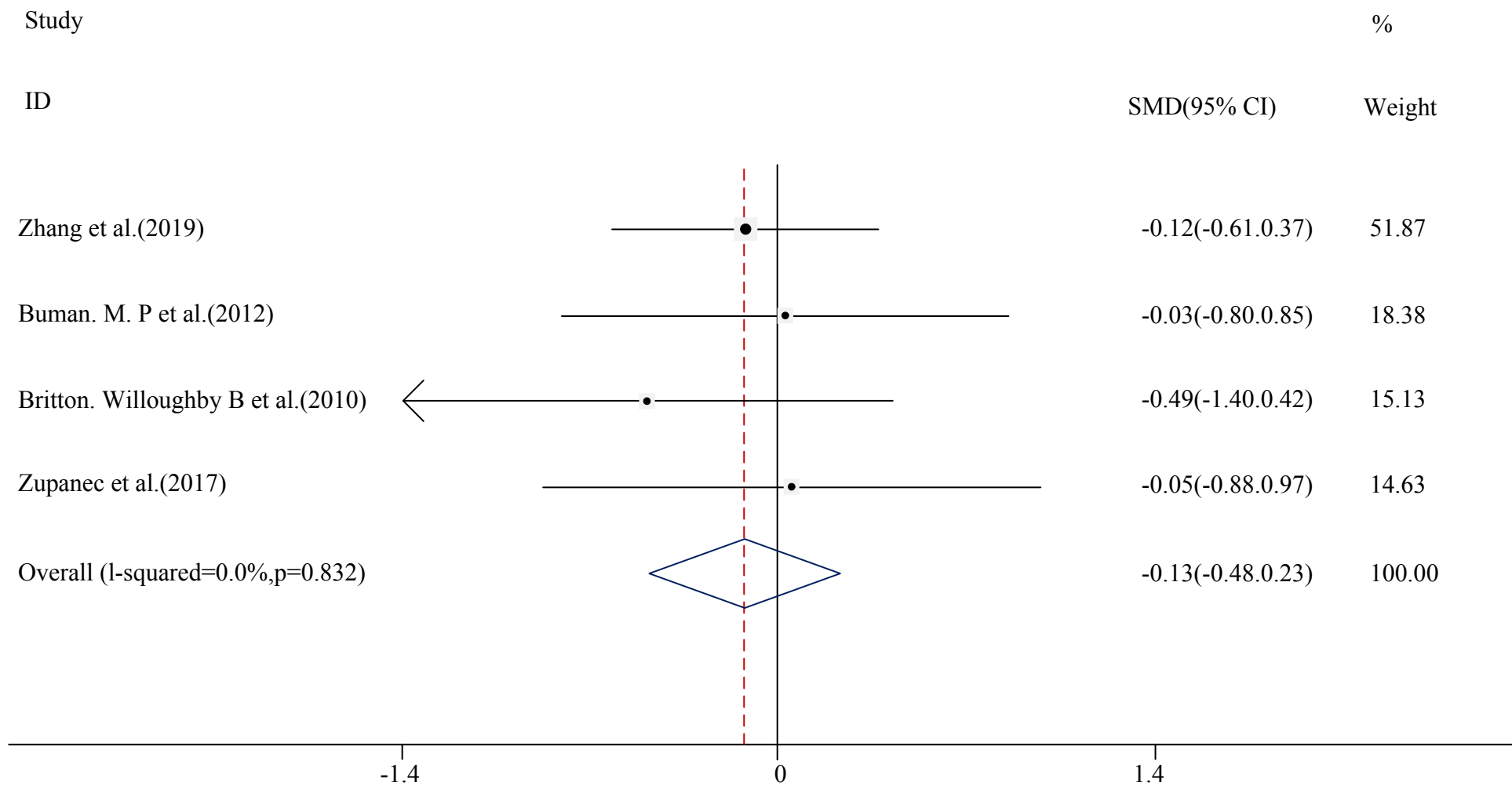

Supplement: Supplementary file 1 [file ijerph-19-10284-s001.zip › Forest plot of PSG outcomes.pdf]
