# Supplementary material for: Mindfulness-Based Movement Intervention to Improve Sleep Quality: A Meta-Analysis and Moderator Analysis of Randomized Clinical Trials
Source: Int J Environ Res Public Health. 2022 Aug 18;19(16):10284. doi: 10.3390/ijerph191610284 (PMC9408303; doi:10.3390/ijerph191610284)
Supplement: Supplementary file 1 [file Risk of bias summary.pdf › Risk of bias summary.pdf]

	Random sequence generation (selection bias)	Allocation concealment (selection bias)	Blinding of participants and personnel (performance bias)	Blinding of outcome assessment (detection bias)	Incomplete outcome data (attrition bias)	Selective reporting (reporting bias)	Other bias
Adler, E. et al 2017	+	+	−	?	+	+	+
Afonso, Rui Ferreira.et al. 2012	+	−	−	−	−	+	+
Agustín et al 2019	+	+	−	+	+	+	+
AKODU AK. et al 2021	+	+	+	+	+	+	−
Aksu, N. T., et al 2018	+	+	−	+	+	+	+
Alexandra Michel. et al 2021	+	?	−	+	+	+	+
Andersen et al. 2013	+	?	−	−	+	+	+
Ann-Charlotte Grahn Kronhed. et al 2020	+	?	−	−	?	+	−
Ashrafinia et al. 2013	+	?	−	−	+	+	+
Bai et al. 2019	+	+	−	−	+	+	+
Biegel, Gina M.et al. 2009	+	−	−	−	+	+	+
Black et al. 2014	+	+	−	+	+	+	+
Blaes et al. 2016	+	?	−	−	+	+	+
Blake et al. 2016	+	+	−	+	+	+	+
Bower, Julienne E.et al. 2012	+	+	−	−	+	+	+
Britton, W. B.et al. 2012	+	+	−	+	+	+	−
Britton, Willoughby B.et al. 2010	+	−	−	+	−	+	−
Bruce et al. 2020	+	+	−	−	+	+	−
Buchanan, D. T., et al 2017	+	+	−	+	−	+	+
Buman, M. P.et al. 2011	+	−	−	−	+	+	+
Buman, M. P.et al. 2011	+	−	−	−	−	+	+
Caldwell, K.et al. 2011	+	−	−	−	+	+	+
Caldwell et al. 2016	+	?	−	+	+	+	+
Carmody, James Francis.et al. 2011	+	−	−	−	+	+	+
Carson, James W.et al. 2009	+	−	−	−	+	+	+
Cetin et al. 2020	+	+	−	−	+	+	−
Chan, A. S.et al. 2012	+	−	−	−	+	+	+
Chan, A. S.et al. 2012	+	−	−	−	−	+	+
Chandwani et al. 2014	+	?	−	−	+	+	+
Chan et al. 2014	+	?	−	−	+	+	−
Chan et al. 2016	+	?	−	+	+	+	+
Chan et al. 2020	+	+	−	−	+	+	−
Chaoul, A., et al 2018	+	?	?	?	−	+	+
Chen, Kuei-Min.et al. 2009	+	−	−	−	+	+	+
Chen, M. C.et al. 2012	+	+	−	−	+	+	+
Curi, V. S., et al 2018	+	?	?	?	+	+	+
de Bruin et al. 2020	+	+	−	−	+	+	−
Dhruva, A.et al. 2012	+	−	−	−	+	+	−
Di Shao. et al 2021	+	+	?	?	+	+	+
Duman, M. and S. Timur Taşhan 2018	−	+	−	+	+	+	+
Ebrahimi, M., et al 2017	+	+	?	?	−	+	+
Emel Sonmezer. et al 2021	+	+	+	+	+	+	+
Encarnación Soriano-Ayala. et al 2020	+	+	+	+	+	+	+
Eric B. Loucks. et al 2021	+	+	+	+	+	+	+
Eric J. Roseen. et al 2020	+	+	+	+	+	+	+
Fan et al. 2019	+	−	−	−	+	+	+
Fang et al. 2015	+	?	−	−	+	+	+
Fritz, T.et al. 2011	+	+	−	−	−	+	+
Frye, B.et al. 2011	+	−	−	−	−	+	+
Garland et al. 2014	+	?	−	+	+	+	+
Greeson et al. 2014	+	+	−	−	+	+	+
Gregoire, C., et al 2017	−	−	?	?	+	+	+
Grensman, A., et al 2018	+	+	−	+	+	+	+
Groessl, E. J., et al 2018	+	+	−	+	+	+	+
Gross, Cynthia R.et al. 2011	+	+	−	−	+	+	+
Guerra et al. 2020	+	+	−	−	+	+	+
Gulhan et al. 2019	+	+	−	+	+	+	−
Habibollahpour et al. 2019	+	+	−	−	+	+	+
Hariprasad et al. 2013	+	?	−	−	+	+	+
Hülsheger et al. 2015	+	?	−	−	+	+	+
Innes, K. E & Selfe, T. K. 2012	+	−	−	−	+	+	+
Innes et al. 2016	+	+	−	−	+	+	+
Irwin, M. R., et al 2017	+	?	−	+	−	+	+
Irwin et al. 2014	+	+	−	+	+	+	+
Jennifer L. Gordon . et al 2021	+	+	−	+	+	+	+
Jennifer L. Huberty. et al 2021	+	+	+	+	+	+	+
Jones, K. D.et al. 2012	+	−	−	−	+	+	+
Jun Jiang. et al 2020	+	+	−	+	+	+	+
Kadriye et al. 2019	+	+	−	−	+	+	−
Kelly Hilcove. et al 2021	+	+	−	+	+	+	+
Kelly Krese. et al 2020	+	+	−	+	?	+	−
Kerekes, N., et al 2017	+	?	−	?	−	+	+
Keyla de Paula Barbosa. et al 2021	+	+	−	+	+	+	+
Kim E. Innes. et al 2020	+	+	−	+	−	+	−
Klatt, M. D.et al. 2009	+	−	−	−	+	+	+
Klose, P., et al 2018	+	+	−	+	−	+	+
Kroz, M., et al 2017	−	−	?	?	−	+	+
Lengacher et al. 2015	+	+	−	+	+	+	+
Lin et al. 2019	+	+	−	+	+	+	−
Lipschitz et al. 2016	+	?	−	−	+	+	+
Liu et al. 2019	+	−	−	−	+	+	+
Lu, J. J., et al 2017	+	?	−	+	−	+	+
Lucinda Kennett. et al 2021	+	?	?	?	+	+	−
Lu et al. 2019	−	−	−	−	+	+	+
Lynch, Mary.et al. 2012	+	+	−	−	−	+	+
Marian E. Papp. et al 2020	+	?	−	+	+	+	−
Maryanna Klatt et al 2017	+	?	?	?	−	+	+
Mayer Bellehsen. et al 2021	+	+	−	+	+	+	−
McQuade, J. L., et al 2017	+	−	−	?	−	+	+
Michael et al. 2019	+	−	−	−	+	+	−
Mustian et al. 2013	+	?	−	+	+	+	+
Nakamura, Yoshio.et al. 2011	+	−	−	−	+	+	+
Nakamura et al. 2013	+	?	−	−	+	+	+
Nguyen, Manh Hung & Kruse, Andreas 2012	+	−	−	−	−	+	+
Ong et al. 2014	+	+	−	−	+	+	+
Özkan, S. A. and G. Rathfisch 2018	+	+	−	−	+	+	+
Parco M. Siu. et al 2021	+	+	+	+	+	+	+
Phenphop Phansuea. et al 2020	+	?	−	−	+	+	+
Rachael B. Smith. et al 2021	+	?	?	?	+	+	+
Reid, K. J.et al. 2010	+	−	−	−	+	+	−
Rshikesan, P. B., et al 2018	+	?	?	?	−	+	+
Sanghee Moon. et al 2020	+	+	+	+	+	+	−
Seyedi Chegeni, P., et al 2018	+	?	+	+	+	+	+
Shree Ganesh H.R.. et al 2021	+	?	?	?	+	+	+
Soon Young Lee. et al 2020	+	+	+	+	+	+	+
Sun et al. 2013	+	?	−	−	+	+	+
Tiffany Low. et al 2020	+	?	−	+	+	+	−
Viviam Vargas Barros. et al 2021	+	?	−	−	+	+	+
Wang, W. C.et al. 2010	+	−	−	+	−	+	+
Wolever, Ruth Q.et al. 2012	+	−	−	−	?	+	+
Yurtkuran, M.et al. 2007	+	−	−	+	+	+	+
Zhang et al. 2015	+	?	−	+	+	+	+
Zhang et al. 2019	+	+	−	+	+	+	+
Zhao Yue. et al 2020	+	+	+	+	+	+	+
Zheng . et al 2019	+	−	−	+	+	+	+
Zhu, D., et al 2018	+	+	−	+	−	+	+
Zülfünaz Ozer,. et al 2021	+	?	−	−	+	+	+
Zülfünaz Ozer & Sebahat Ates 2021	+	+	+	+	+	+	+
Zupanec, S., et al 2017	+	+	+	+	+	+	+
